# Monosodium glutamate is associated with dose-dependent reproductive toxicity and sperm dysfunction in male wistar rats

**DOI:** 10.5935/1518-0557.20250052

**Published:** 2025

**Authors:** Olaleke Abraham Fasasi, Babatunde Oluwaseun Ibitoye, Adesua Emmanuel Ogunmokunwa, Adebanji Modupe Akingbade, Adebukola Olubosede Omolayo

**Affiliations:** 1 Omega Golden Fertility, Lagos, Nigeria; 2 Department of Human Anatomy, Federal University of Technology, Akure, Ondo State, Nigeria; 3 Department of Anatomy, Ekiti State University, Ado Ekiti, Ekiti State, Nigeria; 4 University of Medical Sciences Teaching Hospital, Ondo, Ondo State, Nigeria

**Keywords:** monosodium glutamate, gonadosomatic index, sperm parameters, testicular weight, Wistar rat, low dose male fertility

## Abstract

**Objective:**

Monosodium glutamate (E621) is widely used as a flavor enhancer and is added to many processed foods, often concealed by the label E621. Controversies exist regarding its safety, as most studies use high doses that are challenging to translate to human relevance. This study investigated the effects of chronic low-dose MSG administration, mimicking average daily human usage, on male Wistar rats’ reproductive function.

**Methods:**

Thirty rats were divided into five groups (n=6): Group A (control, 1 ml distilled water) and Groups B, C, D, and E, receiving MSG doses of 30 mg/kg, 100 mg/kg, 300 mg/kg, and 1000 mg/kg body weight, respectively, for 65 days. Parameters measured included body weight, testicular weight, and semen analysis (sperm concentration, morphology, and motility).

**Results:**

Body weight increased significantly in MSG-treated groups, particularly at higher doses (240±20 g for 300 mg/kg and 260±25 g for 1000 mg/kg). Gonadosomatic Index (GSI) rose dose-dependently, while testicular weight declined in higher-dose groups (1.2±0.1g at 1000 mg/kg). Sperm concentration reduced from 80±5 million/ml (control) to 30±2 million/ml (1000 mg/kg), and normal sperm morphology dropped from 76% to 58%. Defects in sperm head, neck, and tail increased with dose, and motility showed a marked decline, with fast progressive sperm decreasing to 39% (1000 mg/kg) and non-motile sperm rising to 51%.

**Conclusions:**

These findings suggest that chronic low-dose MSG exposure negatively impacts male reproductive health, emphasizing the need to assess dietary MSG’s long-term risks.

## INTRODUCTION

Monosodium glutamate (MSG) is a widely used flavor enhancer in various food products, including processed foods, snacks, and seasonings, due to its ability to intensify the umami taste. Its consumption has significantly increased over the years, raising questions about its long-term health implications (Zanfirescu *et al.*, 2019). While MSG has been deemed safe by food regulatory agencies at recommended levels, there is growing concern over its potential adverse effects when consumed in excess or over long periods. Specifically, its impact on metabolic and reproductive health has attracted attention, particularly in males, where reproductive toxicity has been suggested in experimental models (Jubaidi *et al.*, 2019; Nnadozie *et al.*, 2019).

A key mechanism through which MSG exerts its harmful effects is its role in promoting oxidative stress. Oxidative stress arises when the body’s antioxidant defenses are overwhelmed by reactive oxygen species (ROS), leading to cellular and tissue damage. The testes, which are highly sensitive to oxidative stress, are particularly vulnerable. Excessive ROS can impair spermatogenesis, reduce sperm motility, and increase sperm abnormalities, contributing to male infertility (Sengupta *et al.*, 2024; Vahedi Raad *et al.*, 2024). MSG’s ability to trigger oxidative stress has been demonstrated in several studies, which suggest that it can lead to significant reproductive dysfunction in animals. Moreover, MSG has been shown to disrupt hormonal regulation by affecting the hypothalamic-pituitary-gonadal axis, further contributing to reproductive imbalances (Wang *et al.*, 2021; Ogunmokunwa & Ibitoye, 2025).

In addition to its reproductive effects, MSG has been implicated in metabolic disturbances, including weight gain and obesity. Research indicates that MSG can stimulate the hypothalamus, increasing appetite and food intake, leading to hyperphagia and subsequent weight gain (Kayode *et al.*, 2023). This can exacerbate metabolic and oxidative stress, creating a feedback loop that further harms reproductive health. Chronic MSG consumption has also been associated with insulin resistance and dyslipidemia, both of which can negatively impact fertility (Insawang *et al.*, 2012). Given the rising prevalence of MSG in modern diets, these findings raise concerns about its long-term consumption and its possible role in the increasing rates of infertility observed worldwide (López-Miranda *et al.*, 2015; Yang *et al.*, 2023).

Although high doses of MSG have been linked to reproductive toxicity in various animal models, limited studies have examined the effects of chronic low-dose exposure, which better simulate real-life dietary habits. It is crucial to understand whether even modest levels of MSG consumed over time can have significant impacts on reproductive function. Most people consume MSG regularly through processed foods, making it essential to investigate its cumulative effects over long periods. Previous studies that focused on high-dose administration often overlook the potential subtler, but still damaging, effects of chronic exposure at lower levels (Zanfirescu *et al.*, 2019). This study seeks to fill this gap by examining the effect of chronic low-dose MSG on reproductive function in male Wistar rats.

## MATERIAL AND METHODS

### Chemicals

Monosodium glutamate (C_5_H_9_NO_4_Na) with a purity of 99% NT was sold in most open markets under the license of Ajinomoto Co. Inc., Tokyo, Japan. A stock solution was prepared by dissolving 60g of MSG crystals in 1000 ml of distilled water. The dose schedule was so adjusted that the amount of MSG administration per animal was as per their respective weight.

### Animals Study

This study was performed on 30 healthy, mature male Wistar rats, aged 8-10 weeks and weighing 150-220g. Animals were obtained from the Animal House of Biochemistry Department, Federal University of Technology, Akure. Animals were housed in ventilated cages (60 cm × 80 cm × 18 cm) kept in a well-ventilated room with temperatures ranging between 22 and 25°C and maintained under standardized conditions away from any stressful conditions with 12/12 light and dark cycle with free access to humidity and were fed standard rodent chow (Top Feed Nigeria Limited), with constant access to tap water.

### Animal Grouping and Treatment

The animals were divided into five groups:

Group A (Control): Administered 1 ml distilled water daily.

Group B: Administered 30 mg/kg body weight MSG daily.

Group C: Administered 100 mg/kg body weight MSG daily.

Group D: Administered 300 mg/kg body weight MSG daily.

Group E: Administered 1000 mg/kg body weight MSG daily.

The MSG solutions were prepared fresh daily and administered orally for 65 consecutive days via oral gavage.

### Experimental Procedure and Animal Sacrifice

The body weight of each animal was recorded weekly throughout the study period. At the end of the experimental period, all animals were anaesthesized with 5mg/Kg of ketamine via intramuscular route. The testes were carefully dissected through abdomen and weighed. The GSI was calculated as the ratio of testes weight to body weight, expressed as a percentage.

### Semen Collection and Analysis

Semen samples were collected from the cauda epididymis immediately after dissection. The contents of epididymis were diluted using the HTF medium (1:200 v:v). After shaking once, 10 µl of the specimen was transferred to a Neubauer hemocytometer and placed for 5 min in the humidified chamber. The number of counted cells in five squares with a light microscope under a 20× microscope objective was expressed as the number of sperm/ ml. For the evaluation of sperm motility percentage, one drop of the specimen was placed on an incubated glass slide (37°C) and covered with a lamella. The percentage of motile cells was recorded in 10 different microscopic fields under a 20× microscope objective. The evaluation of sperm viability was carried out by adding 10 µl of 0.50% eosin Y and nigrosin staining solution into an equal volume of the specimen. The examination was done on the slides incubated for 2 min at room temperature. The head of dead sperm cells was stained pink while the head of live cells appeared pale. One hundred randomly chosen spermatozoa were evaluated under a 100× microscope objective.

### Statistical Analysis

Data were expressed as mean ± standard error of the mean (SEM) and analyzed using one-way ANOVA. The assumptions of ANOVA were verified: independence was ensured by random group assignment, normality was confirmed using the Shapiro-Wilk test (*p*>0.05), and homogeneity of variances was validated with Levene’s test (*p*>0.05). Significant differences were assessed with Tukey’s post hoc test, and *p*<0.05 was considered statistically significant. Analyses were performed using GraphPad Prism version 8 (GraphPad Software, Inc., USA).

## RESULTS

The effects of chronic administration of MSG on body weight, GSI, and sperm parameters were assessed in male Wistar rats.

### Body Weight

**Figure 1:**
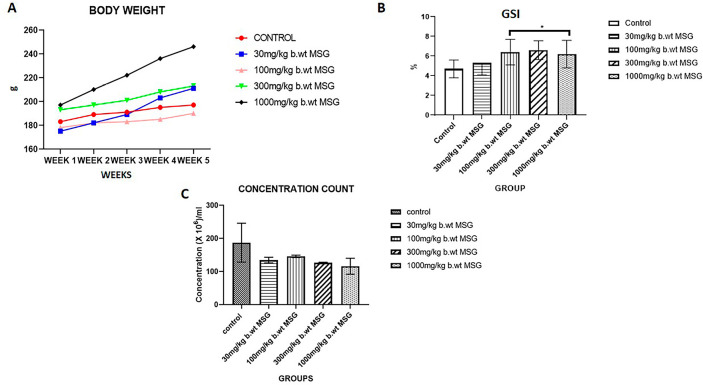
A) Changes in body weight over 5 weeks in male Wistar rats administered varying doses of monosodium glutamate (MSG). Values represent mean±SEM (n=6). Significant differences compared to the control group are denoted (*p<0.05). (B) Gonadosomatic Index (GSI) of male Wistar rats following 65 days of MSG exposure. Data shown as mean±SEM (n=6). Significant differences compared to the control group are denoted (*p<0.05). (C) Sperm concentration counts in male Wistar rats exposed to different MSG doses for 65 days. Values represent mean±SEM (n=6). Significant differences compared to the control group are denoted (*p<0.05).

Figure 1A shows the changes in body weight over the experimental period. Rats treated with MSG exhibited a dose-dependent increase in body weight compared to the control group. The highest increase was observed in the 1000 mg/kg MSG group (*p*<0.05), indicating significant weight gain associated with higher MSG doses.

### GSI

The GSI values across the groups are presented in Figure 1B. A significant increase in GSI was observed in the 300 mg/kg and 1000 mg/kg MSG groups compared to the control group (*p*<0.05). These findings suggest that MSG induces body weight gain while reducing relative testicular weight, particularly at higher doses.

### Sperm Concentration

As shown in Figure 1C, sperm concentration decreased progressively with increasing MSG doses. The highest dose group (1000 mg/kg MSG) showed the lowest sperm concentration compared to the control group, with significant differences observed (*p*<0.05).

### Sperm Morphology

**Figure 2:**
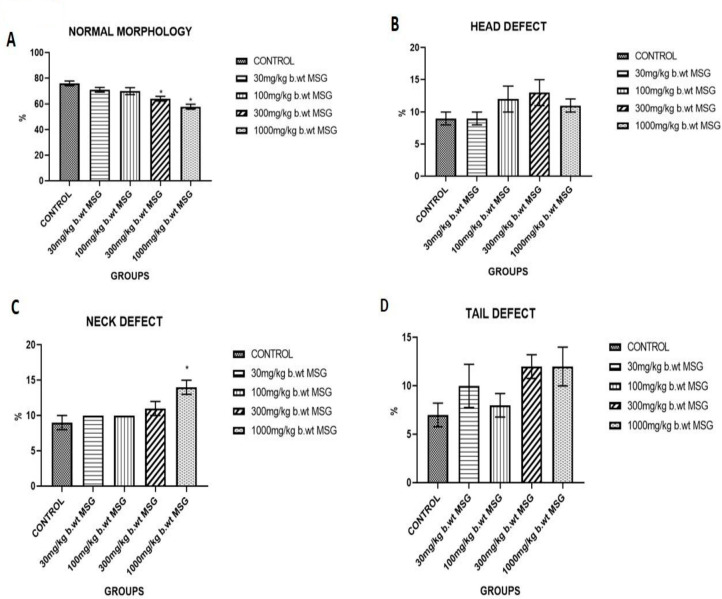
(A) Percentage of normal sperm morphology in male Wistar rats treated with different doses of MSG for 65 days. (B) Percentage of sperm head defects in MSG-treated groups. (C) Percentage of sperm neck defects observed in different MSG exposure groups. (D) Percentage of sperm tail defects across treatment groups. Values represent mean±SEM (n=6). Significant differences compared to the control group are denoted (*p<0.05).

Figure 2A demonstrates a dose-dependent decline in normal sperm morphology with MSG administration. The control group exhibited the highest percentage of normal sperm morphology (approximately 76%), whereas the 1000 mg/kg MSG group showed a significant reduction to 58% (*p*<0.05).

### Sperm Defects

Figures 2B-2D present the distribution of sperm abnormalities, including head, neck, and tail defects. Sperm head defects increased significantly in the 300 mg/kg and 1000 mg/kg MSG groups (*p*<0.05). Similarly, the percentage of sperm with neck and tail defects rose significantly in the higher-dose MSG groups, with the most pronounced abnormalities in the 1000 mg/kg MSG group (*p*<0.05).

### Sperm Motility

**Figure 3:**
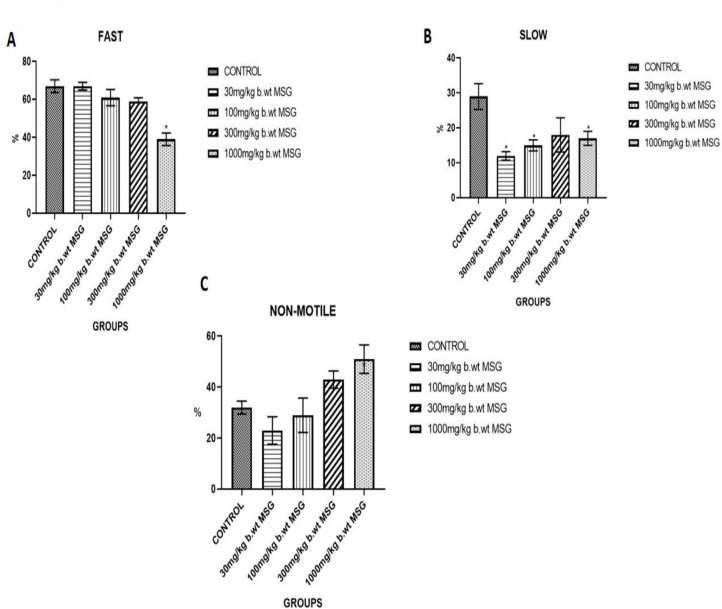
(A) Percentage of fast progressive motility in spermatozoa from male Wistar rats exposed to different MSG doses. (B) Percentage of slow progressive motility in spermatozoa across treatment groups. (C) Percentage of non-motile sperm observed in each treatment group. Values represent mean±SEM (n=6). Significant differences compared to the control group are denoted (*p<0.05).

Figure 3 illustrates the impact of MSG on sperm motility. Fast progressive motility (Figure 3A) decreased significantly with increasing MSG doses, with the 1000 mg/kg group exhibiting the lowest motility (*p*<0.05). In contrast, the percentage of non-motile sperm (Figure 3C) increased in a dose-dependent manner, with the 1000 mg/kg group having the highest percentage of non-motile sperm (*p*<0.05).

## DISCUSSION

The GSI, measuring the ratio of testicular weight to body weight, offers valuable insight into the reproductive health effects of MSG exposure. The GSI values across the groups reveal a dose-dependent effect of MSG on both body and testicular weight (Figure 1A-B). Group A (Control) has the lowest GSI, indicating a normal balance between body and testicular weight, reflecting no external disruptions. In healthy animals, the GSI remains low, which is consistent with a lack of oxidative stress or testicular damage (Manna & Jain, 2015; Suleiman *et al.*, 2020). In Group B (30 mg/kg MSG), the slight increase in GSI suggests that even low doses of MSG start affecting body and testicular weight. At this dose, the body weight may have increased due to MSG’s ability to stimulate appetite, promoting mild hyperphagia through hypothalamic stimulation (Elefteriou *et al.*, 2003; Anderson *et al.*, 2018; Kayode *et al.*, 2023) while testicular weight remains relatively stable, indicating minimal reproductive toxicity. Group C (100 mg/kg MSG) shows a further increase in GSI, implying more significant body weight gain. The increase is likely due to MSG-induced adiposity and metabolic disturbances, which have been linked to increased body weight (Kayode *et al.*, 2023). However, testicular weight might have started to decline slightly due to oxidative stress affecting testicular tissue, as reported by López-Miranda *et al.* (2015). Group D and E (300 mg/kg MSG and 1000 mg/kg respectively) exhibits very high GSI, suggesting both an increase in body weight and a decrease in testicular weight (Figure 1A-B). Research shows that at this dose, MSG leads to oxidative stress, which impairs Leydig cells and causes testicular atrophy (Wang *et al.*, 2021; Ogunmokunwa & Ibitoye, 2025). This combination of testicular mass reduction and body weight gain explains the sharp increase in GSI.

The sperm concentration data shows a dose-dependent reduction in sperm count with MSG, demonstrating its toxic effect on male reproductive health even at low dose (Figure 1C). Group A (Control) shows the highest sperm concentration, representing normal spermatogenesis and healthy reproductive function without any exposure to MSG (Haddad *et al.*, 2021). Group B (30 mg/kg MSG) reveals a decline in sperm concentration, reflecting early effects of MSG on the reproductive system. Studies have indicated that even low doses of MSG can induce oxidative stress in the testes, which affects the integrity of spermatogenic cells, leading to reduced sperm production (Oluwole *et al.*, 2024). Group C (100 mg/kg MSG) exhibits a slight increase in sperm concentration compared to Group B but still remains significantly lower than the control group. This could be a temporary adaptive response to moderate oxidative stress. However, research suggests that sustained exposure to moderate doses of MSG leads to Leydig cell dysfunction, which affects testosterone levels and impairs spermatogenesis (Das & Ghosh, 2010; Haddad *et al.*, 2021). The findings are also consistent with studies reporting chronic MSG exposure as a cause of persistent oxidative stress and damage to the seminiferous tubules, where sperm is produced (Abd-Elkareem *et al.*, 2021). In summary, the data on sperm concentration (Figure 1C) confirms that higher doses of MSG result in more severe testicular damage and sperm production impairment, consistent with findings from various studies (Farombi & Onyema, 2006; Igwebuike *et al.*, 2011; Abd-Elkareem *et al.*, 2021; Oluwole *et al.*, 2024).

The sperm morphology data reflects the effects of increasing doses of MSG on reproductive health, with significant declines in normal sperm morphology and increases in sperm defects (head, neck, and tail) as the dosage increases (Figure 2A-D). Group A (Control) shows the highest percentage of normal sperm (76%), indicating healthy spermatogenesis without exposure to MSG. Untreated animals typically display higher percentages of normal sperm, reflecting optimal reproductive health (Eweka *et al.*, 2011; Igwebuike *et al.*, 2011). Group B (30 mg/kg MSG) and Group C (100 mg/kg MSG) show slight declines in normal sperm morphology (71% and 70%, respectively). These reductions could be due to the early toxic effects of MSG, particularly oxidative stress, which has been shown to impair sperm morphology and function at even moderate doses (Oluwole *et al.*, 2024). Group D (300 mg/kg MSG) and Group E exhibit even more significant reductions in normal sperm morphology (64% and 58%, respectively) (Figure 2A). This trend is consistent with previous studies that report a dose-dependent increase in sperm abnormalities following high doses of MSG, which compromises sperm quality due to oxidative damage and apoptosis of germ cells (Abd-Elkareem *et al.*, 2021). Group A (Control) has the lowest percentage of head defects (9%), reflecting the normal variability in sperm morphology. Group B (30 mg/kg) and Group C (100 mg/kg) show no significant changes in head defects (9% and 12%), suggesting that at lower doses, MSG has a relatively mild effect on head morphology. However, in Group D (300 mg/kg) and Group E, there is an increase in head defects (13% and 11%), reflecting more severe damage to sperm development at higher MSG doses (Figure 2B). This could be attributed to oxidative stress, which disrupts normal DNA packaging in sperm heads, leading to morphological abnormalities (Wang *et al.*, 2021). Group A shows minimal neck defects (9%), which are considered within the normal range. There is a gradual increase in neck defects in Groups B (10%) and C (10%), with a more pronounced rise in Group D (11%) and Group E (14%) (Figure 2C). Neck defects can be associated with cytoskeletal damage during spermatogenesis, which is known to be exacerbated by oxidative stress and testicular cell damage, as seen with increasing MSG exposure (Omolaoye *et al.*, 2025). Tail defects follow a similar pattern, with Group A showing the lowest percentage (7%). MSG exposure causes a noticeable increase in tail defects in Group B (10%), Group C (8%), and especially Group D and E (12% each) (Figure 2D. Tail defects are commonly associated with impaired motility, and research has shown that high doses of MSG impair sperm motility by damaging microtubule structures within the tail (Oluwole *et al.*, 2024).

The sperm motility data highlights the impact of MSG on the progression (fast, slow, and non-motile sperm) across the various dose groups (Figure 3A-C). Sperm motility is a critical determinant of male fertility, and the observed changes indicate the detrimental effects of MSG on sperm function. The control group shows the highest percentage (67%) of fast-progressing sperm, which is expected in healthy animals. Fast motility is essential for the sperm to reach and fertilize the egg, and this group, with no MSG exposure, displays optimal sperm motility, reflecting healthy reproductive function (Kianifard *et al.*, 2021). There is no significant reduction (67%) in fast-progressing sperm among the animals in Group B (30 mg/kg MSG), suggesting that minimal exposure does not immediately impact sperm motility. However, earlier research has shown that even at low doses, MSG can lead to oxidative stress, but it may not yet manifest in significant motility changes (Omolaoye *et al.*, 2025). A slight reduction in fast motility (61%) is observed in Group C (100 mg/kg), indicating that moderate MSG exposure begins to impair sperm progression. This could be attributed to the oxidative damage affecting sperm tail structures, which are essential for motility (Oluwole *et al.*, 2024). Group D (300 mg/kg MSG) shows a further decline in fast sperm motility (59%), reflecting more severe damage to sperm function. This is likely due to mitochondrial damage and ROS (reactive oxygen species) generation caused by MSG, which interferes with the energy required for rapid sperm movement (Igwebuike *et al.*, 2011; Kianifard *et al.*, 2021). Group E shows the sharpest drop in fast progression (39%), indicating a significant impairment of sperm motility (Figure 3A). This decline is consistent with high-dose MSG exposure, which induces testicular damage and disrupts the sperm’s ability to move efficiently (Hamza & Al-Harbi, 2014; Kianifard *et al.*, 2021). Group A (29%) exhibits a healthy balance between fast and slow-progressing sperm. Slow motility is typical in a portion of sperm but should not dominate in healthy reproductive conditions. Group B (12%) shows a noticeable reduction in slow motility, suggesting that the majority of sperm in this group are either fast-progressing or non-motile. The drop in slow motility at this dose reflects the early toxic effects of MSG, which may selectively affect slower-moving sperm first (Omolaoye *et al.*, 2025). Group C (15%) indicates that while some sperm maintain slow motility, the majority are either fast-progressing or suffering from impaired motility due to oxidative stress (Oluwole *et al.*, 2024; Ogunmokunwa & Ibitoye, 2025). Group D (18%) and Group E (17%) show a marginal increase in slow progression, reflecting the shift as more sperm lose their capacity for fast motility, gradually moving towards either slow progression or becoming non-motile (Figure 3B). Group A (32%) shows a typical percentage of non-motile sperm, as even in healthy conditions, a certain proportion of sperm are non-motile. In Group B (30 mg/kg MSG), this percentage decreases to 23%, suggesting that initial exposure to MSG does not significantly impair motility; however, early signs of MSG toxicity may still affect sperm function. Research indicates that even low doses of MSG can induce oxidative stress, potentially compromising sperm motility (Hamza & Al-Harbi, 2014). In Group C (100 mg/kg MSG), the percentage of non-motile sperm rises to 29%, indicating a cumulative effect of moderate MSG exposure that damages sperm motility machinery. This is further reflected in Group D (300 mg/kg MSG), where non-motile sperm increase to 43%, highlighting greater testicular damage and significant oxidative stress that impacts motility (Omolaoye *et al.*, 2025). Group E (1000 mg/kg MSG) shows the highest percentage of non-motile sperm at 51%, indicating severe testicular dysfunction due to prolonged high levels of MSG (Figure 3C). At this stage, oxidative damage can severely impact ATP production in mitochondria, critical for sperm movement (Wang *et al.*, 2021; Ogunmokunwa & Ibitoye, 2025).

## CONCLUSION

This study demonstrates that chronic exposure to low doses of MSG has a dose-dependent detrimental effect on reproductive function in male Wistar rats. As MSG dosage increased, significant disruptions were observed in body weight, testicular weight, and sperm parameters, including sperm concentration, morphology, and motility. The reduction in testicular weight and sperm quality, especially at higher doses, suggests that MSG may induce oxidative stress and metabolic disturbances, leading to testicular atrophy and impaired spermatogenesis. These findings highlight the potential reproductive risks associated with chronic MSG consumption, even at low doses, which could have implications for human health, particularly in populations with high dietary MSG intake. Further research is needed to explore the underlying mechanisms of MSG toxicity on reproductive health and to determine whether these effects are reversible or persist over time.
